# Ezh2-mediated epigenetic modification is required for allogeneic T cell-induced lupus disease

**DOI:** 10.1186/s13075-020-02225-9

**Published:** 2020-06-05

**Authors:** Yuxuan Zhen, Roger D. Smith, Fred D. Finkelman, Wen-Hai Shao

**Affiliations:** 1 Division of Immunology, Allergy and Rheumatology, Department of Internal Medicine, University of Cincinnati, Cincinnati, OH 45267 USA; 2 Department of Pathology, University of Cincinnati, Cincinnati, OH 45267 USA; 3 Division of Immunobiology, Cincinnati Children’s Hospital Medical Center, Cincinnati, OH 45229 USA

**Keywords:** SLE, cGVHD, Ezh2, H3Kme, GSK503, GC B cells, T_FH_ cells, OVA-immunization

## Abstract

**Background:**

The mechanisms involved in the pathogenesis of autoimmune disorders, including systemic lupus erythematosus (SLE), have not been fully elucidated. Some of these mechanisms involve epigenetic regulation of gene expression. The histone methyltransferase Ezh2 contributes to epigenetic regulation of gene expression, is highly expressed in germinal center (GC) B cells and follicular T helper (T_FH_) cells, and may be involved in lupus pathogenesis.

**Methods:**

The murine bm12 model of lupus-like chronic graft versus host disease (cGVHD) was induced by intra-peritoneal injection of negatively isolated allogeneic CD4^+^ T cells. Lupus-like disease development was monitored by ELISA determination of serum anti-dsDNA and anti-chromatin antibody titers. Immune cell activation and Ezh2 expression were evaluated by flow cytometry and Western blotting.

**Results:**

Decreased autoantibody production and GC formation are observed when Ezh2-deficient CD4^+^ T cells are used instead of wild-type (WT) to induce cGVHD and when mice that receive allogeneic WT donor T cells to induce cGVHD are treated with GSK503, an Ezh2-specific inhibitor. In the bm12 cGVHD model, WT donor T cells are normally fully activated 1 week after infusion into an allogeneic host, exhibit a T_FH_ cell (PD-1^hi^/CXCR5^hi^) phenotype with upregulated Ezh2, and activate B cells to form germinal centers (GCs). In contrast, Ezh2-deficient donor T cells generate fewer T_FH_ cells that fail to activate B cells or promote GC formation. Despite similar T-independent, LPS-induced B cell responses, OVA-immunized CD4.Ezh2-KO mice had a skewed low-affinity IgM phenotype in comparison to similarly treated WT mice. In addition, early after OVA immunization, more CD4^+^ T cells from B6.CD4.Ezh2-KO mice had a CD44^lo^/CD62L^lo^ phenotype, which suggests arrested or delayed activation, than CD4^+^ T cells from ovalbumin-immunized B6.WT mice.

**Conclusion:**

Ezh2 gene deletion or pharmacological Ezh2 inhibition suppresses autoantibody production and GC formation in bm12 lupus-like cGVHD and decreases affinity maturation and isotype switching in response to immunization with a T cell-dependent antigen. Ezh2 inhibition may be useful for the treatment of lupus and other autoimmune disorders.

## Background

Systemic lupus erythematosus (SLE), an autoimmune disorder, is characterized by a spectrum of autoantibodies that target multiple cellular components. The etiology and pathogenesis of SLE are not yet fully elucidated [1] and the molecular mechanisms underlying disordered T and B cell activation and differentiation in SLE are still poorly defined. Emerging evidence has illustrated the importance of epigenetic dysregulation in the pathogenesis of SLE; for example, autoreactive T and B cells in SLE patients have been shown to have altered patterns of DNA methylation and histone modification [2].

Enhancer of zester homolog 2 (Ezh2) is a histone methyltransferase (HMT) that catalyzes trimethylation of histone H3 at lysine 27 (H3Kme3) and acts primarily as a gene silencer as part of the polycomb repressive complex 2 (PRC2) [3]. Ezh2 also catalyzes methylation of non-histone proteins and has the ability to recruit nuclear receptors to specific promoters [4–6]. In general, Ezh2 controls cell proliferation, differentiation, and function [7]. Despite their primary function as gene silencers, Ezh2 and H3Kme3-marked histones are critical for proper T and B cell lineage development and activation [4, 7, 8]. Mature naive T cells express low levels of Ezh2, but rapidly upregulate Ezh2 upon TCR ligation and alloantigen stimulation [9]. Similarly, Ezh2, although important for VDJ recombination in pre-B cells, is undetectable in mature lymph node B cells, but is considerably expressed in GC B cells [10] and is required for plasma cell differentiation and maximum Ab secretion [11].

GSK503 inhibits Ezh2’s methyltransferase activity with > 200-fold more selectivity for Ezh2 than Ezh1 and > 4000-fold more selectivity for Ezh2 than for other histone methyltransferases. GSK503 displays favorable pharmacokinetics in mice, in which it reduces GC B cell numbers and splenocyte H3K27me3 levels by inhibiting the catalytic function of Ezh2 [12, 13]; similar effects are observed in vitro.

Bm12-induced chronic graft-versus-host disease (cGVHD) is initiated by injecting mice with MHC class II incompatible CD4^+^ T cells or unfractionated spleen cells. It results in a chronic syndrome characterized by the production of high titers of a spectrum of autoantibodies similar to those seen in human SLE, accompanied by some of the pathological manifestations of SLE [14]. The cGVH syndrome is indistinguishable between the transfer of C57BL/6 cells into bm12 mice (B6 → bm12 transfer) and bm12 → B6 transfer, though the B6 → bm12 transfer is less well studied. The autoantibodies produced in this model of cGVHD come entirely from the recipient mouse’s B cells [15]. Using this model, we investigated the role of Ezh2 in the development of B6 → bm12 cGVHD. To our surprise, Ezh2-deficient CD4^+^ T cells failed to induce autoantibody production in this mouse model. Deficiency of Ezh2 in CD4^+^ T cells failed to alter T-independent B cell responses, but diminished the T-dependent B cell responses to immunization with ovalbumin. Pharmacological inhibition of Ezh2, like genetic Ezh2 deficiency, ameliorated autoantibody production in the bm12 model of lupus-like cGVHD, raising the possibility that Ezh2 could serve as a therapeutic target.

## Methods

### Experimental mice

Ezh2^fl/fl^ (022616, JAX), C57BL/6 (000664, JAX), and CD4^cre^ (022071, JAX) mice were originally purchased from Jackson Laboratory and maintained in our colony. B6.bm12 mice bearing CD45.1 marker were provided by Dr. Edith Janssen (Cincinnati Children’s Hospital Medical Center) and maintained in our colony. B6.Ezh2^fl/fl^ mice were backcrossed onto a C57BL/6 background (> 8 generations) and then crossed with CD4^Cre^ mice to generate mice with Ezh2 deficiency in CD4^+^ T cells (designated as CD4.Ezh2-KO mice). Eight- to 10-week-old age-matched female mice were used in experiments. Experimental protocols were approved by the University of Cincinnati’s Committee on Use and Care of Animals (IACUC).

### Chronic GVH diseases induced by CD4 T cells

Chronic GVHD was induced in bm12 host mice as previously described [16]. In brief, CD4^+^ T cells were purified from B6.WT or B6.CD4.Ezh2-KO spleens using the CD4^+^ T cell negative isolation kit (Miltenyi Biotec, Auburn, CA). Chronic GVHD was induced by intra-peritoneal injection of 1 × 10^7^ purified CD4^+^ T cells into host B6.bm12 mice. For GSK503 treatment group, cGVHD was induced by injecting donor T cells into B6.bm12 hosts 2 days after the initiation of GSK503 treatment (125 mg/kg/day i.p.). Autoimmune disease development was followed by serum levels of autoantibodies against dsDNA and chromatin.

### LPS injection and OVA immunization

Mice were injected i.p. with either 200 μl of LPS (from *E. coli* 055:B5; Sigma-Aldrich), or with 300 μg of OVA (Sigma-Aldrich, absorbed onto alum). Mouse sera were collected at different time points and stored at − 20 °C for ELISA. Single spleen cell suspensions were stained for CD4, CD44, and CD62L and processed for analysis by flow cytometry.

### ELISA

For anti-dsDNA ELISA, 96-well plates were pre-coated with L-lysine (0.01%, Sigma-Aldrich, St. Louis, MO) for 1 h; plates were then washed and incubated with dsDNA overnight. For anti-chromatin and total IgG ELISA, 96-well plates were directly incubated with chicken chromatin and anti-mouse IgG (1 μg/ml) overnight, respectively. Mouse sera (1:250 diluted) were then added into each well of the 96-well plate and incubated overnight at 4 °C. Plates were washed and incubated with alkaline phosphatase-conjugated goat anti-mouse IgG (0.1 μg/ml, Fc-specific, Jackson ImmunoResearch Lab, West Grove, PA) for 2 h at room temperature. Plates were washed again and p-nitrophenyl phosphate substrate (Sigma-Aldrich, St. Louis, MO) was added.

For anti-OVA ELISA, plates were coated with OVA (10 μg/ml in PBS) overnight at 4 °C. Plates were washed once with distilled water, then blocked with 1% BSA in PBS overnight at 4 °C, and incubated with various dilutions of serum for 2 h at 37 °C. After 3 washes with buffer (0.05% Tween-20 in PBS), biotinylated goat anti-mouse IgM, or IgG1, IgG2c, IgG2b, IgG3, and IgG antibodies (Southern Biotechnology Associates, Birmingham, AL) diluted 1:5000 in blocking buffer, was added for 1 h at 37 °C. Plates were washed again 3 times and the alkaline phosphate substrate p-nitrophenyl phosphate (Sigma, St. Louis, MO) was added. The OD was measured at 405 nm using the BioTek microplate reader (Winooski, VT).

### Immunofluorescent staining

Spleen sections (4 μm) were fixed in acetone for 10 min and then blocked with 5% BSA in TBS buffer with 0.1% Tween for 20 min. Sections were then incubated with 1:100 dilutions of anti-mouse antibodies (IgD and GL-7) from BD Biosciences (San Jose, CA) and (anti-Ezh2, anti-rabbit IgG-Alex 488, anti-rabbit IgG-Rhodamine red) from Cell Signaling Technology (Beverly, MA). Images were acquired using a Leica DMi8 fluorescence microscope (Buffalo Grove, IL) and analyzed with the LAX S software developed by Leica Microsystems Inc.

### Flow cytometry analysis

Single spleen cell suspensions were obtained and Fc receptors were blocked with 2.4G2 (100 μg/ml) for 30 min on ice. Cells were then incubated with antibodies as indicated in the figure legends. For phenotypic analysis, T cells were gated on CD4 and analyzed for T_FH_ (CXCR5^+^, PD-1^+^) and T_eff_ (CD44^hi^, CD62L^lo^) markers. B cells were gated on CD19 and analyzed for GC B cell markers (GL-7^+^, CD95^+^). Data were acquired with a BD LSR II flow cytometer (BD Biosciences) and analyzed using FlowJo Software 10.4 (San Carlos, CA).

### Western blot analysis

Spleen samples were homogenized in RIPA buffer (Santa Cruz Biotechnology, CA) with proteinase and phosphatase inhibitors (Roche, Indianapolis, IN). Proteins were separated by SDS-PAGE, transferred to a PVDF membrane (Millipore, Billerica, MA), incubated with anti-Ezh2 antibody (Cell Signaling Technology, Boston, MA), and subsequently with secondary anti-rabbit IgG antibodies labeled with HRP, both in blocking buffer (1% dry milk). Proteins were visualized with enhanced chemiluminescence substrate (Fisher Scientific, Hampton, NH).

### Statistical analysis

Western blot data were analyzed using ImageJ software (NIH, Bethesda, MD). Intensity differences between groups were tested using the Mann-Whitney *U* test. Data are shown as medians with interquartile range. Comparison of two means was analyzed using the two-tailed unpaired Student *t* test. All statistical analyses were carried out using Prism 7 software (GraphPad, San Diego, CA). Results were considered significant at *p < 0.05, **p < 0.01, ***p < 0.001.

## Results

### Ezh2 deficient T cells fail to induce lupus-like cGVHD

**Fig. 1:**
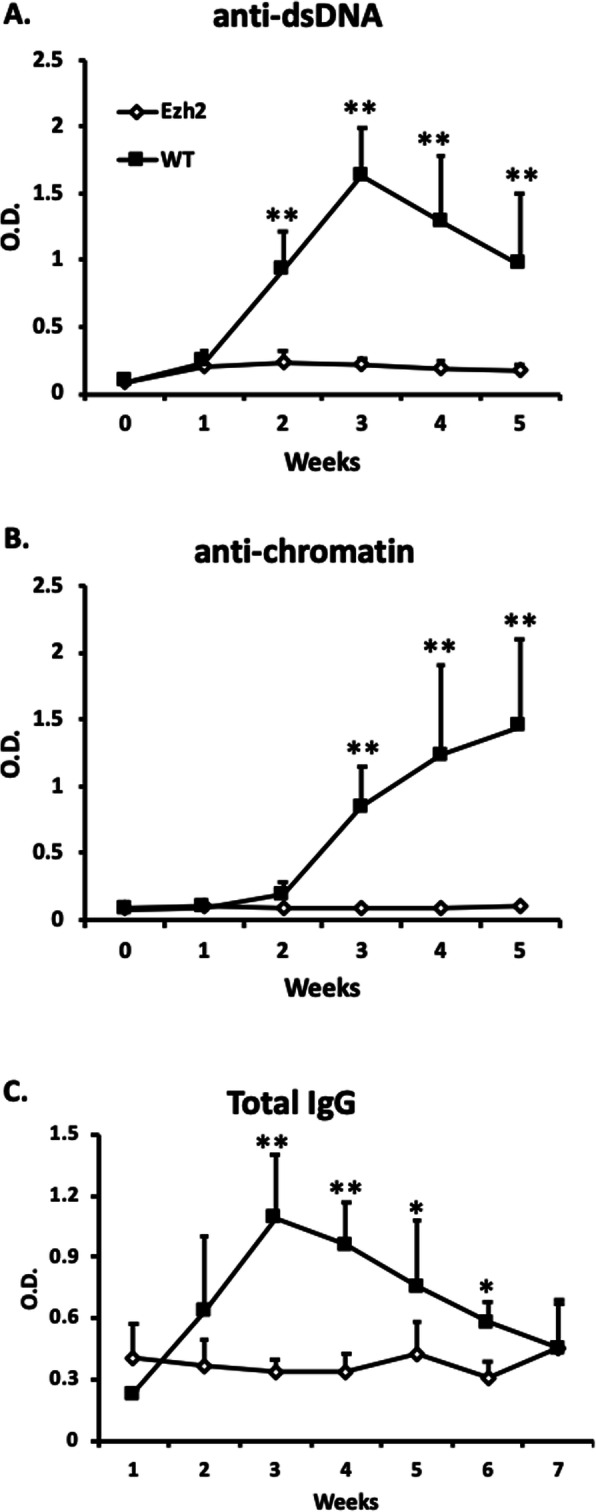
Dependence of cGVHD-associated autoimmunity on CD4^+^ T cell Ezh2. bm12 mice (5 mice/group) were injected ip with 1 × 10^7^ negatively selected CD4^+^ T cells from C57BL/6 (WT) or B6.CD4.Ezh2-KO (Ezh2) mice. Mouse sera were collected weekly. Total IgG and autoantibodies against dsDNA and chromatin were analyzed by ELISA [16, 19]. Data are representative of four repeats. Error bars show standard deviations. Student’s *t* test, *p < 0.05; **p < 0.01

The mouse model of cGVHD is characterized by the production of autoantibodies. Allogeneic donor CD4^+^ T cells drive polyclonal activation of recipient B cells, which increases total serum IgG and promotes specific autoantibody production [16–18]. To study the role of Ezh2 in allogeneic T cell-mediated loss of B cell tolerance, cGVHD was induced in bm12 host mice by administering negatively purified CD4^+^ T cells from either WT mice or CD4.Ezh2-KO mice. As expected, B6 → bm12 transfer induced anti-dsDNA and anti-chromatin Abs and increased total IgG levels in the serum of bm12 recipient mice (Fig. 1a–c). None of these effects were induced by CD4.Ezh2-KO → bm12 transfer. These findings suggest an important role of T cell Ezh2 in the development of lupus-like cGVHD.

### Diminished T and B cell activation in CD4.Ezh2-KO T cell-induced cGVHD

**Fig. 2:**
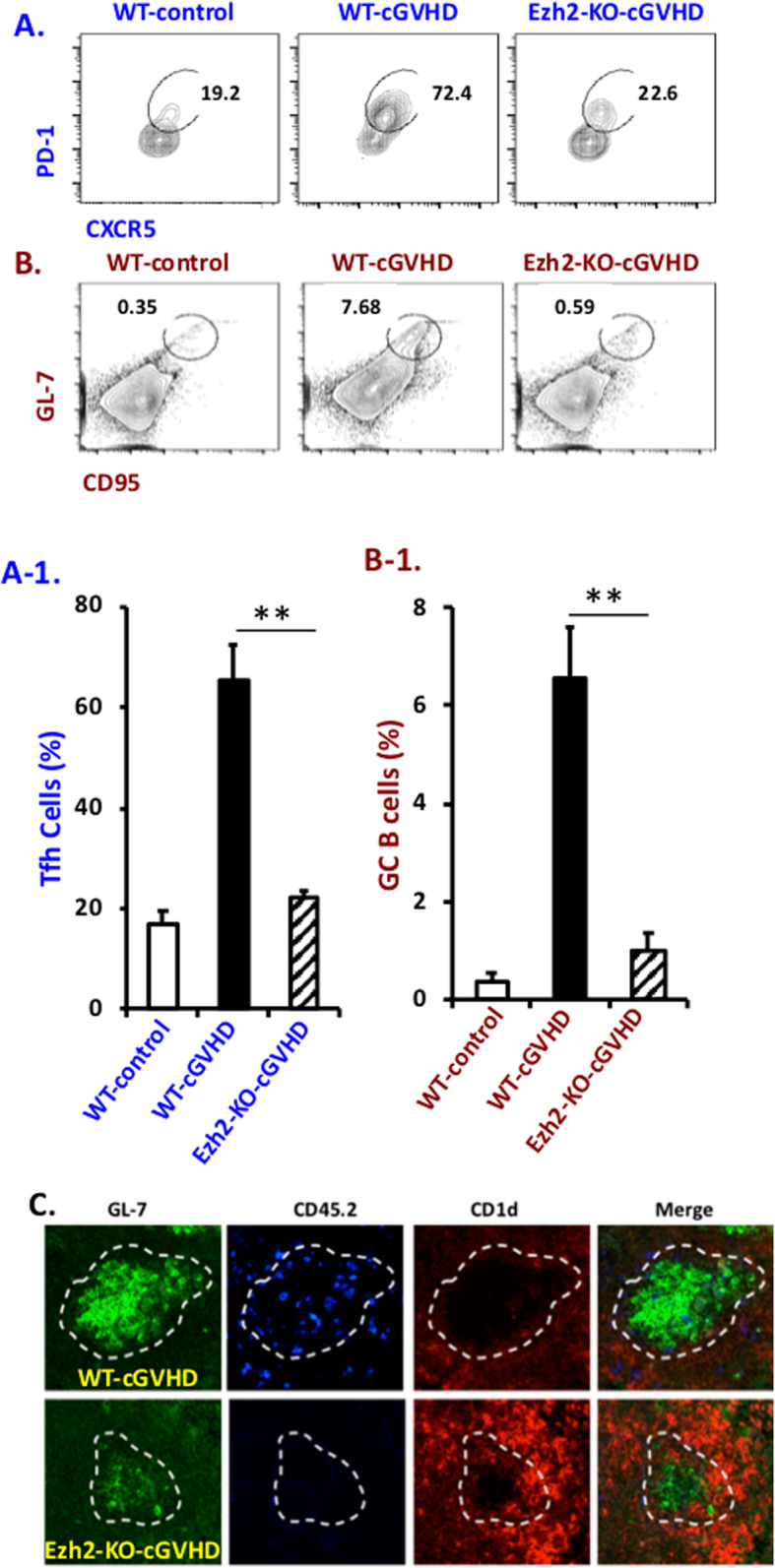
CD4^+^ T cell Ezh2 is important for T_FH_ cell and GC B cell responses in cGVHD. Lupus-like cGVHD was induced in bm12 host mice as in Fig. 1. Single cell splenocyte preparations were stained with markers for **a** T_FH_ cells (CD4^+^/CXCR5^+^/PD-1^+^) and **b** GC B cells (CD19^+^/GL-7^+^/CD95^+^). Percentages of splenic T_FH_ and GC B cells are shown in A1 and B1, respectively. Student’s *t* test, **p < 0.01. **c** Spleen sections (4 μM) were blocked and incubated with antibodies against mouse GL-7 (green), CD45.2 (blue, donor CD4^+^ T cell-specific), and CD1d (red). Images are representative of at least 3 sections from individual mice in each experiment and were evaluated in 2 independent experiments

cGVHD is induced by cognate interaction of donor CD4^+^ T cells with the allogeneic MHC class II on recipient B cells. By 1 week after their encounter with bm12 host B cells, donor CD4^+^ T cells display an activated phenotype, characterized by decreased CD62L and increased CD44 [20]. Some of these T cells had migrated into the GC center and differentiated into follicular T helper cells (T_FH_, CXCR5^hi^, PD-1^hi^, Fig. 2a). Interaction with these T cells activates naïve B cells to differentiate into GC B cells, undergo receptor editing and maturation, and further differentiate into cells that have a GC B cell phenotype (GL-7 and CD95 (Fas) double positive, Fig. 2b). However, very few CD4.Ezh2-KO T cells enter GCs and develop a T_FH_ phenotype (Fig. 2a). As a result, smaller GCs were observed in the spleens of bm12 mice that received CD4.Ezh2-KO rather than WT T cells (Fig. 2b, c). Thus, CD4^+^ T cells lacking Ezh2 are incapable of full activation and migration into the GC and have a considerably reduced ability to induce B cell activation, maturation, and autoantibody production.

### Ezh2-expressing cells localized to GCs

**Fig. 3:**
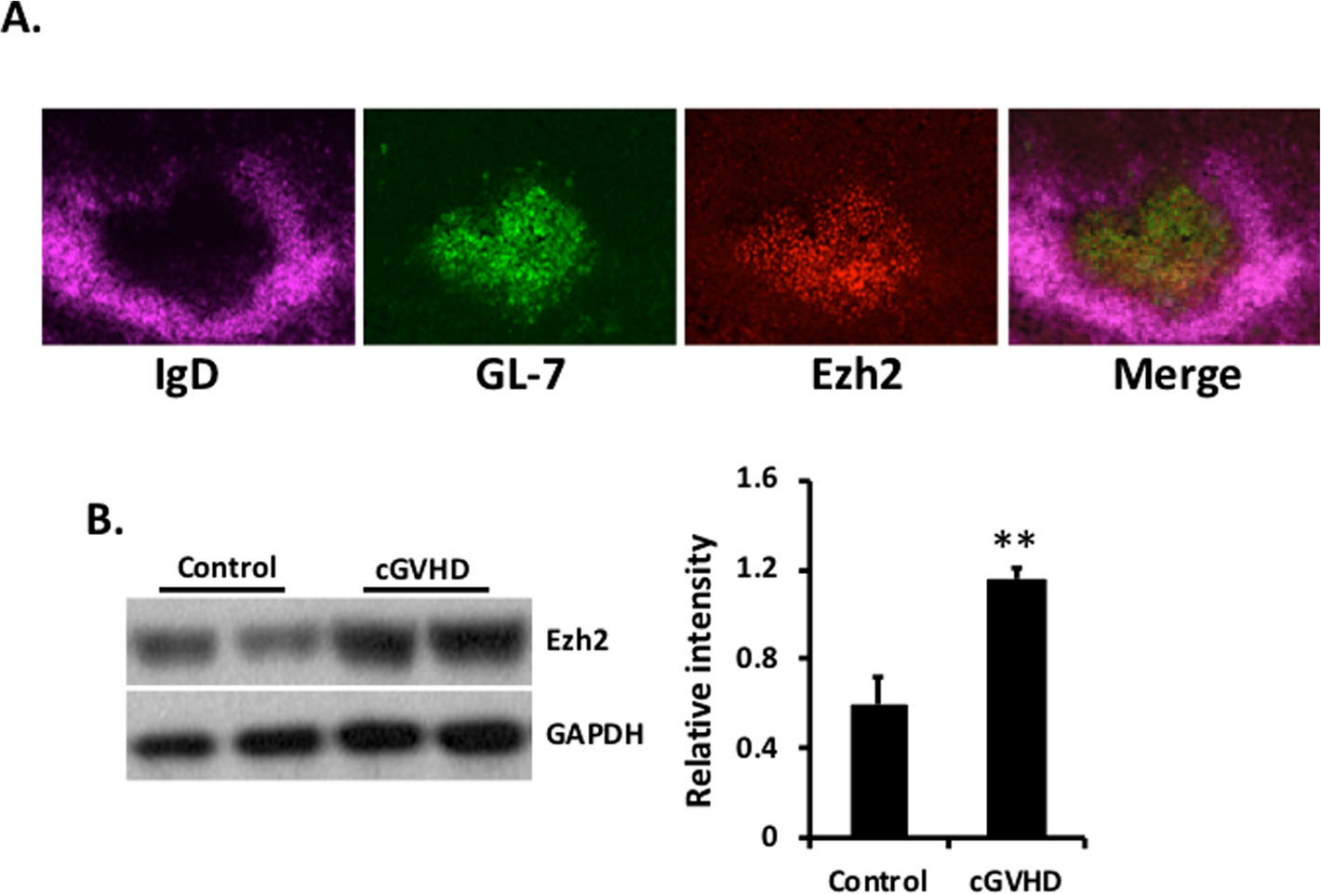
Ezh2 is predominantly expressed in GC cells. cGVHD was induced in bm12 recipients of CD4^+^ T cells from B6.WT or B6.CD4.Ezh2-KO mice. **a** Spleen sections from a WT T cell recipient were stained for IgD, GL-7, and Ezh2. **b** Spleen samples from experimental mice were homogenized and blotted with antibodies against mouse Ezh2 and GAPDH (loading control). Densitometry analyses are shown. Bars show the median and interquartile range. **p < 0.01, Mann-Whitney *U* test

Ezh2 has been reported to be upregulated in T cells upon TCR allo-stimulation [21] and to be required for B cell development and GC B cell activation [10, 11]. To gain insight into the splenic cell populations that express Ezh2, we stained spleen sections from cGVHD bm12 host mice with fluorochrome-labeled mAbs to IgD, Ezh2, and GL-7 (a GC cell marker). As shown in Fig. 3a, Ezh2 was highly upregulated in splenic GCs in cGVHD mice. The upregulation of Ezh2 was further confirmed by Western blot (Fig. 3b). Upregulation of Ezh2 during cGVHD raised the possibility that suppression of Ezh2 might decrease the immunological sequelae of cGVHD, including its lupus-like features.

### Genetic depletion of Ezh2 from CD4^+^ T cells suppresses T-dependent, but not T-independent B cell responses

**Fig. 4:**
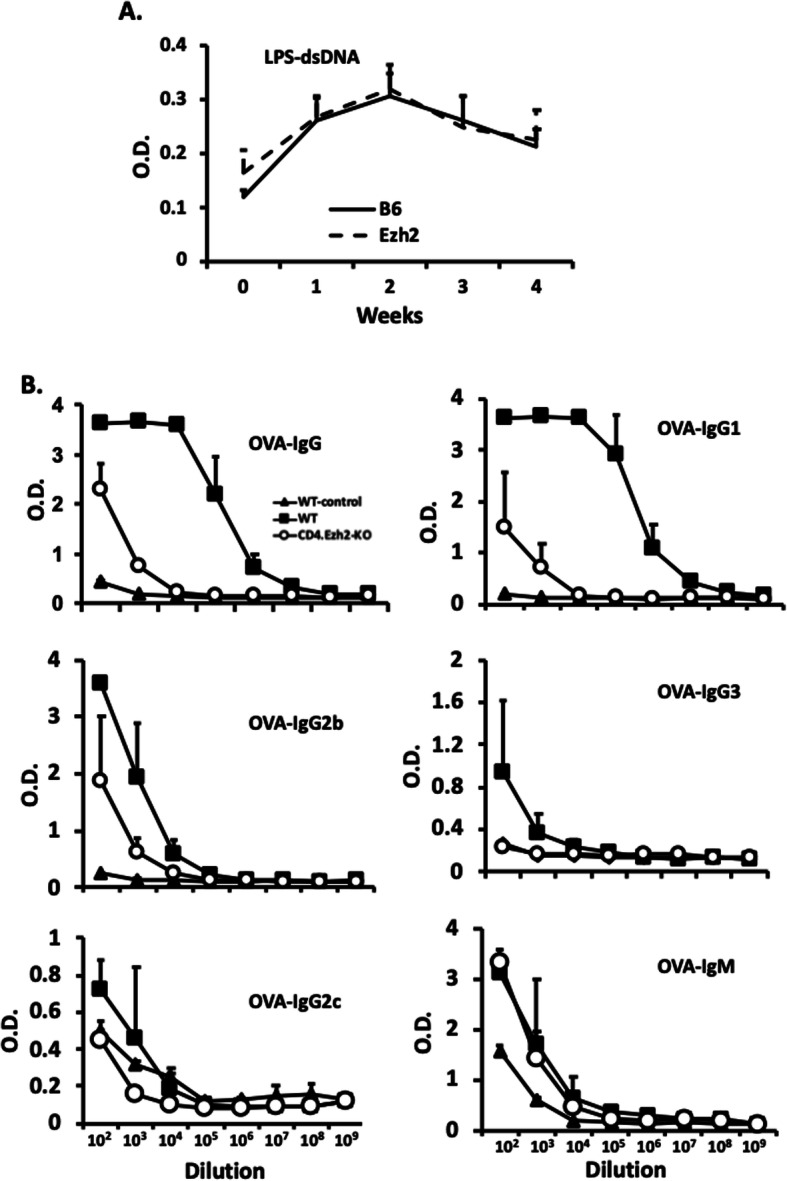
T cell Ezh2 deficiency suppresses T cell-dependent, but not T-independent Ab responses. **a.** Mice were injected with 200 μg of LPS i.p. at day 0. Serum levels of anti-dsDNA were detected by ELISA as in Fig. 1. **b** CD4.Ezh2-KO and B6.WT mice (*n* = 5) were injected i.p. with 300 μl of OVA adsorbed to alum. Sera were collected on day 14 and serum levels of isotypes of anti-OVA antibody were measured by ELISA. Solid triangle, WT-control group, untreated; solid square, WT group, OVA-treated; open circle, CD4.Ezh2-KO group, OVA-treated

The lack of autoimmunity and polyclonal B cell activation in CD4.Ezh2-KO T cell-induced cGVHD could be due to T cell unresponsiveness, T cell failure to stimulate B cell responses, or a failure of activate autoreactive B cells. To investigate this, we evaluated the Ezh2 requirement for T cell-dependent and T cell-independent B cell responses. B cells from B6.WT mice and B6.CD4.Ezh2-KO mice generated equivalent anti-DNA Ab responses to the T cell-independent Ag and polyclonal activator, LPS (Fig. 4a). In contrast, B6.CD4 Ezh2 mice made markedly deficient IgG (especially IgG1), but not IgM responses to the T cell-dependent immunogen alum-adsorbed ovalbumin (OVA) (Fig. 4b). Thus, CD4^+^ T cell Ezh2 is important for T cell-dependent, but not T cell-independent B cell Ab responses.

### Ezh2 deficiency is associated with T cell activation arrest

**Fig. 5:**
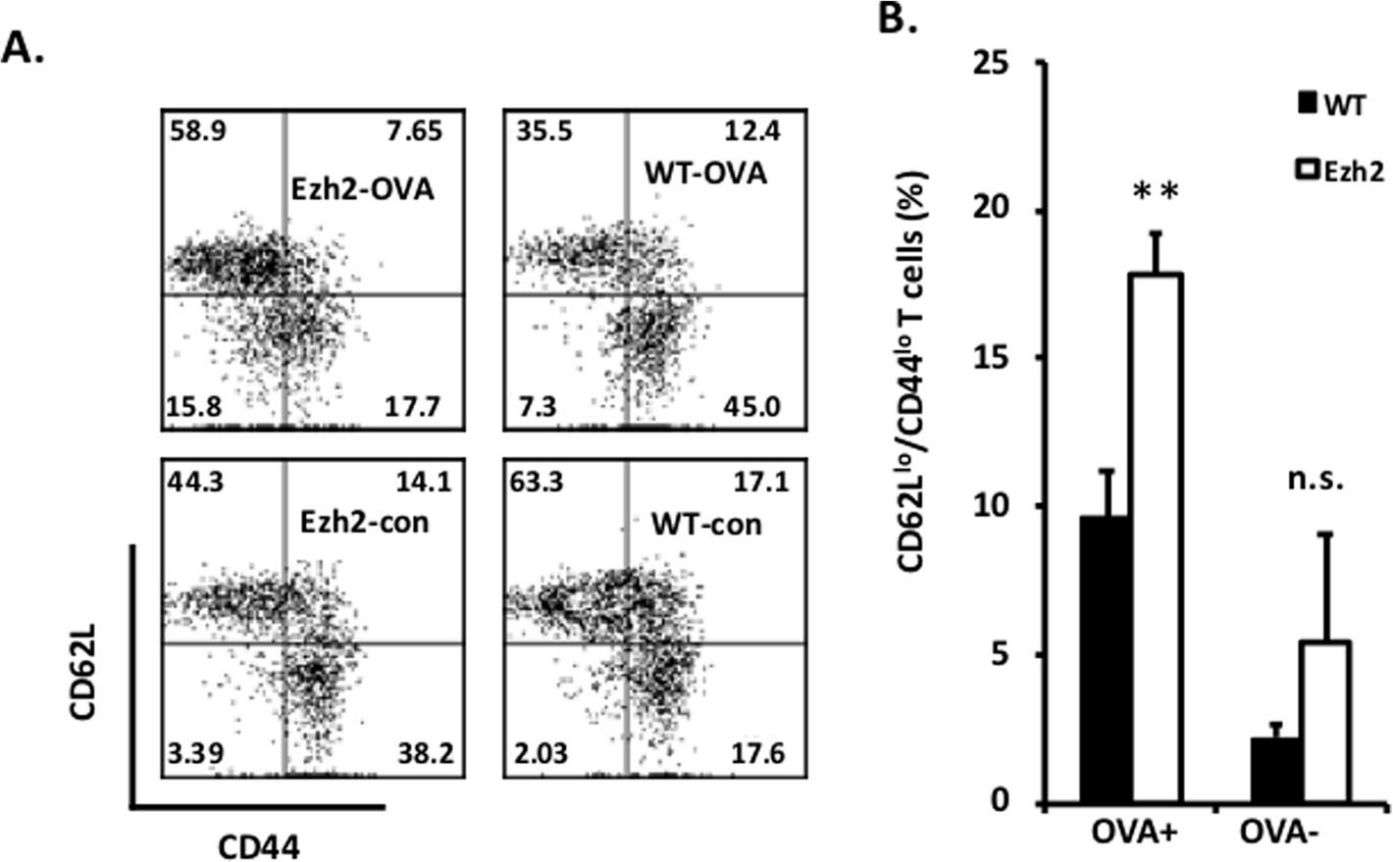
Immunization-induced activation is arrested at an early stage in CD4.Ezh2-KO T cells. Mice were injected with OVA adsorbed to alum as in Fig. 4b. Two weeks later, spleen cells were stained with fluorochrome-conjugated Abs against CD4, CD44, and CD62L and analyzed by flow cytometry. **a** Representative data are shown from two experiments. **b** Percentages of CD62L^lo^ CD44^lo^ T cells are shown in the bar graph. Statistical significance was determined using Student’s *t* test, **p < 0.01

Ezh2 expression has been shown to be upregulated by TCR stimulation by alloantigens and to be required for T cell activation and lineage commitment [3, 22]. We have demonstrated CD4^+^ T cell activation and maturation (CD44 upregulation and CD62L downregulation) in bm12 cGVHD mice [20]. To determine if CD4+ T cell activation is impaired by Ezh2 deficiency, we immunized B6.WT and B6.CD4.Ezh2-KO mice with OVA/alum and evaluated CD4^+^ T cell populations 2 weeks later. Immunization induced ~ 30% of CD4^+^ T cells from B6.WT mice to shift from a naïve (CD44^**−**^/CD62L^**+**^) phenotype to an effector (CD44^**+**^/CD62L^**−**^) phenotype (Fig. 5a, right panels). In contrast, following immunization of B6.CD4.Ezh2-KO mice, a considerable percentage of CD4^+^ T cells remained at the early activation stage (CD62L^lo^/CD44^lo^) and there was a decrease in the percentage of CD62^lo^/CD44^hi^ cells (Fig. 5a, left panels). This suggests that Ag-stimulation induces incomplete activation (activation arrest) for CD4^+^ T cells from B6.CD4.Ezh2-KO mice. A similar failure to fully activate Ag-stimulated CD4+ T cells may underlie defective T cell-dependent B cell activation in the bm12 cGVHD model.

### A small molecule Ezh2 inhibitor suppresses cGVHD

**Fig. 6:**
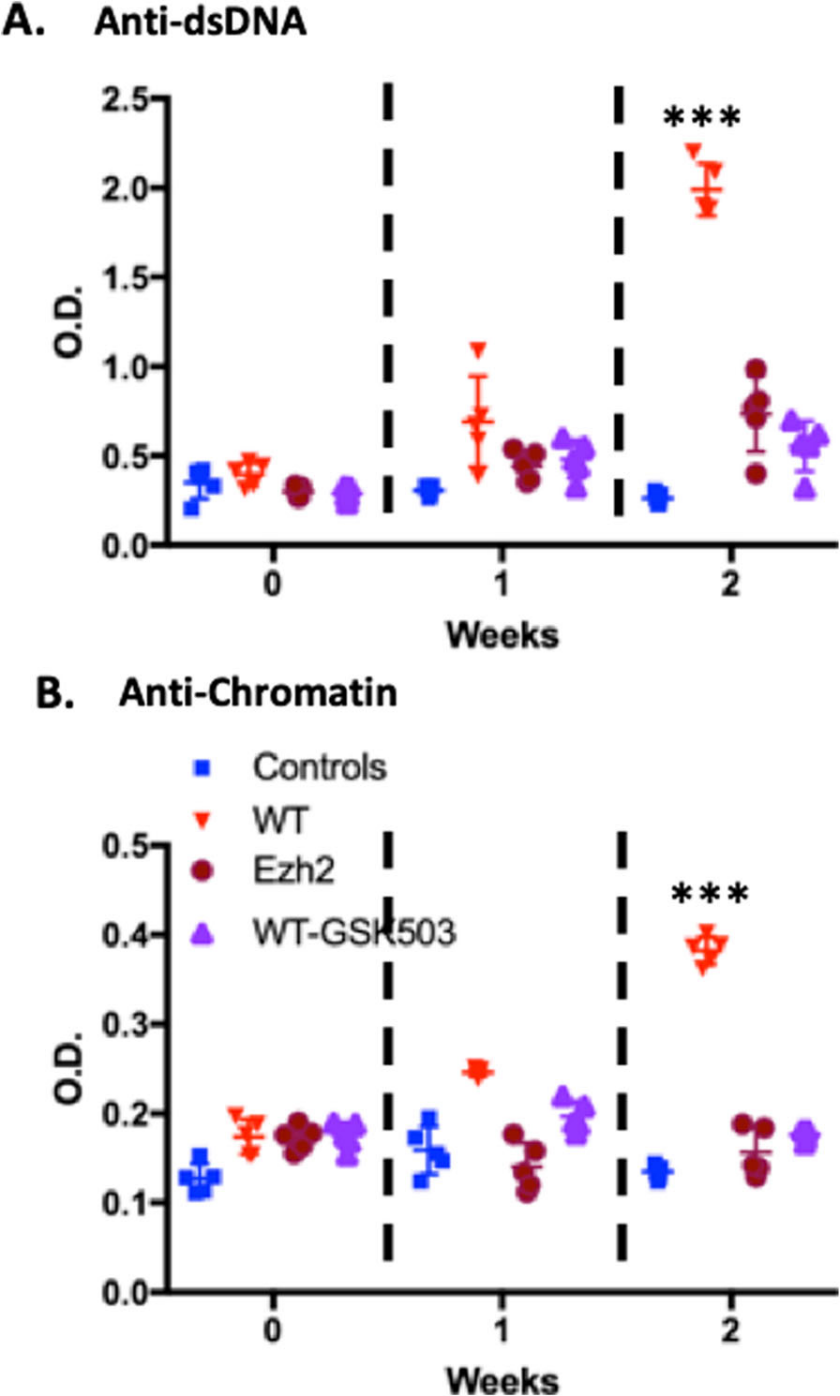
GSK503 decreases autoantibody production in bm12 cGVHD. Lupus-like cGVHD was induced as in Fig. 1. GSK503 (125 mg/kg/day) was injected daily i.p [24]. starting 2 days before the induction of cGVHD. Anti-dsDNA and anti-chromatin autoantibody ELISAs were performed [14]. Five mice/group. Controls are non-cGVHD mice. Comparison between groups was assessed using the parametric Student *t* test. ***p < 0.001

The decreased severity of bm12 cGVHD when Ezh2 genetically deleted in donor T cells and previous observations that Ezh2 is upregulated in GC T and B cells [10, 23] suggested that pharmacological inhibition of Ezh2 might suppress lupus-like features of bm12 cGVHD. We tested this hypothesis by evaluating the ability of GSK503, a small molecule Ezh2 inhibitor, to suppress disease in our cGVHD model when treatment is initiated 2 days before donor T cell injection. Mice were followed with serum levels of anti-dsDNA and anti-chromatin antibody. As expected, serum anti-dsDNA and anti-chromatin Ab levels increased in 2 weeks in bm12 host mice injected with WT, but not Ezh2-deficient CD4^+^ T cells (Figs. 1 and 6). Importantly, the increase in autoantibody levels in bm12 hosts injected with WT B6 CD4^+^ T cells was totally prevented by the GSK503 treatment (Fig. 6). These observations suggest that agents, such as GSK503, that specifically target Ezh2 may be useful for the treatment of SLE autoimmunity.

## Discussion

The etiology and pathogenesis of SLE are not fully known. Current therapies can improve outcomes for lupus but are nonspecific and often associated with multiple adverse side effects [25]. A potential new approach has emerged from studies that illustrate the importance of epigenetic dysregulation in SLE pathogenesis [26]. Several studies have shown that autoreactive T and B cells in patients with SLE and mice with SLE-like disease have evidence of altered patterns of DNA methylation and histone modification [2] with Ezh2 upregulation in B and T cells [7]. These observations about Ezh2 are relevant because of its involvement in gene regulation, including T cell differentiation and lineage commitment [3]. Our study suggests that Ezh2-mediated epigenetic alteration is a primary promoter of autoAb production in the bm12 cGVHD model of lupus. Importantly, Ezh2 was shown to be upregulated exclusively in the GC when autoimmune reactivity is elicited (Fig. 3). Inhibition of Ezh2 by GSK503 significantly reduced autoantibody production in our lupus model.

Ours is not the first study to suggest that epigenetic regulation might have therapeutic value in SLE. Previous studies have shown that treatment with a global histone methylation inhibitor, DZNep, suppresses lupus development in MRL/lpr mice [27, 28]. However, DZNep targets many methyltransferases in addition to Ezh2 [29]. Consequently, the mechanism of DZNep inhibition of murine lupus remains to be defined. Furthermore, DZNep treatment has adverse effects in animal models [30]. In contrast, GSK503 inhibits Ezh2 much more strongly than it inhibits other histone methyltransferases, yet still effectively blocks bm12-induced lupus-like cGVHD in mice (Fig. 6) without obvious adverse effects. Although bm12 cGVHD-induced autoimmunity has considerable limitations as a model for spontaneous human SLE, the rapid onset of autoimmune disease in the mouse model (development of lupus-like features and autoAbs in 2–5 weeks) provides a considerable advantage for studies of mechanisms of pharmacologic inhibition of the development of lupus-like disease [15, 18]. Although this model should also be useful for determining whether Ezh2 can suppress established autoimmunity, studies in spontaneous lupus models, such as the NZB/W mice [31], will be needed prior to clinical trials in human SLE patients.

Clinical trials of Ezh2 inhibitors, however, are already underway. Ezh2 is not only involved in autoimmunity; its expression and contribution to cellular dysregulation have been demonstrated in numerous studies of malignant cells [7]. Consequently, Ezh2 inhibitors have been actively investigated in studies of cancer pathogenesis and are being evaluated in cancer clinical trials (https://clinicaltrials.gov). Results from those studies should accelerate the design and completion of complementary trials in SLE patients.

Ezh2 acts primarily as a transcriptional repressor of multiple genes. Recent studies, however, indicate that in addition to Ezh2’s canonical repression of transcription through histone trimethylation, JAK3- or Akt-mediated Ezh2 phosphorylation makes Ezh2 a methyltransferase-independent transcriptional activator that upregulates a set of genes involved in DNA replication, the cell cycle, and biosynthesis [3, 5]. Phosphorylation of Ezh2 also leads to STAT3 activation through lysine methylation, at least in stem cells [6, 32]. Furthermore, GSK126, a selective small molecule Ezh2 inhibitor that is structurally related to GSK503 [13], has been shown to decrease levels of phosphorylated STAT3 (pSTAT3) in glioma cells that resemble stem cells [6]. These observations may be relevant to SLE, inasmuch as increased STAT3 activity is associated with lupus and lupus nephritis [33, 34]. This may be tied to the roles of specific cytokines in lupus pathogenesis. For example, elevated IL-17 levels have been identified in blood and renal tissues of lupus patients [35] and a positive correlation between IL-17 expression and pStat3 has been reported in these patients [34]. pSTAT3 also promotes the expression of another cytokine in lupus T cells that has been associated with SLE: IL-10 [36]. Despite this, non-canonical activation of Ezh2 has not yet been investigated in the setting of autoimmune pathogenesis. Based on these observations, it is reasonable to hypothesize that Ezh2 activity promotes lupus pathogenesis through non-canonical activation of the STAT3 pathway as well as through its methyltransferase activity. Evaluation of this hypothesis should provide a useful basis for future studies.

Ezh2 is upregulated in multiple cell types in lupus patients [27] and is known to be important in T cell differentiation and function [21, 37] and in GC B cell proliferation [23]. Indeed, a recent study demonstrated that Ezh2 promotes B cell differentiation into plasmablasts through suppression of the BTB and CNC homolog 2 (BACH2) (doi:10.1002/ART.41208). Our immunofluorescence staining revealed predominant splenic upregulation of Ezh2 in the GC (Fig. 3), and our functional studies demonstrated an Ezh2 requirement for GC maturation and T and B cell activation/differentiation. Consistent with this, CD4^+^ T cells lacking Ezh2 were incapable of differentiation into T_FH_, which led to significant reduction of GC B cell numbers and autoAb production in the bm12 cGVHD model. Although our bm12 GVHD studies focused on suppression of Ezh2 activity in CD4^+^ T cells as a way to suppress humoral autoimmunity, suppression of Ezh2 activity in B cells might also be effective, inasmuch as Ezh2 is required for B cell activation and differentiation into antibody-secreting cells (ASC) [11].

## Conclusion

SLE is a complex, multifactorial disease of unknown precise origin. Current therapies can suppress lupus, but are nonspecific and associated with multiple adverse side effects. Data presented here indicate that Ezh2 gene deletion or pharmacological Ezh2 inhibition suppresses autoantibody production and GC formation in bm12 induced lupus-like cGVH disease. This observation, if applicable to spontaneous lupus is likely to have important implications for the treatment of active SLE: an agent such as an Ezh2 inhibitor that blocks both B and T cell differentiation, might be particularly efficacious for the treatment of SLE and other autoimmune disorders.

## Data Availability

The datasets used and/or analyzed during the current study are available from the corresponding author on reasonable request.

## Abbreviations

B6: C57BL/6 J
cGVHD: Chronic graft versus host disease
Ezh2: Enhancer of zester homolog 2 gene
GC: Germinal center
H3Kme3: Histone H3 at lysine 27 trimethylation
SLE: Systemic lupus erythematosus

## References

[1] Liu Z, Davidson A (2012). Taming lupus-a new understanding of pathogenesis is leading to clinical advances. Nat Med.

[2] Xiao G, Zuo X (2016). Epigenetics in systemic lupus erythematosus. Biomed Rep.

[3] He S, Xie F, Liu Y, Tong Q, Mochizuki K, Lapinski PE, Mani RS, Reddy P, Mochizuki I, Chinnaiyan AM (2013). The histone methyltransferase Ezh2 is a crucial epigenetic regulator of allogeneic T-cell responses mediating graft-versus-host disease. Blood.

[4] Burmeister T (2016). EZH2: a pleiotropic protein. Blood.

[5] Yan J, Li B, Lin B, Lee PT, Chung TH, Tan J, Bi C, Lee XT, Selvarajan V, Ng SB (2016). EZH2 phosphorylation by JAK3 mediates a switch to noncanonical function in natural killer/T-cell lymphoma. Blood.

[6] Kim E, Kim M, Woo DH, Shin Y, Shin J, Chang N, Oh YT, Kim H, Rheey J, Nakano I (2013). Phosphorylation of EZH2 activates STAT3 signaling via STAT3 methylation and promotes tumorigenicity of glioblastoma stem-like cells. Cancer Cell.

[7] Gan L, Yang Y, Li Q, Feng Y, Liu T, Guo W (2018). Epigenetic regulation of cancer progression by EZH2: from biological insights to therapeutic potential. Biomark Res.

[8] Melnick A (2012). Epigenetic therapy leaps ahead with specific targeting of EZH2. Cancer Cell.

[9] He SB, Zhou H, Zhou J, Zhou GQ, Han T, Wan DW, Gu W, Gao L, Zhang Y, Xue XF (2015). Inhibition of EZH2 expression is associated with the proliferation, apoptosis, and migration of SW620 colorectal cancer cells in vitro. Exp Biol Med (Maywood).

[10] Beguelin W, Popovic R, Teater M, Jiang Y, Bunting KL, Rosen M, Shen H, Yang SN, Wang L, Ezponda T (2013). EZH2 is required for germinal center formation and somatic EZH2 mutations promote lymphoid transformation. Cancer Cell.

[11] Guo M, Price MJ, Patterson DG, Barwick BG, Haines RR, Kania AK, Bradley JE, Randall TD, Boss JM, Scharer CD (2018). EZH2 represses the B cell transcriptional program and regulates antibody-secreting cell metabolism and antibody production. J Immunol.

[12] Zingg D, Debbache J, Schaefer SM, Tuncer E, Frommel SC, Cheng P, Arenas-Ramirez N, Haeusel J, Zhang Y, Bonalli M (2015). The epigenetic modifier EZH2 controls melanoma growth and metastasis through silencing of distinct tumour suppressors. Nat Commun.

[13] Kaniskan HU, Martini ML, Jin J (2018). Inhibitors of protein Methyltransferases and Demethylases. Chem Rev.

[14] Shao WH, Gamero AM, Zhen Y, Lobue MJ, Priest SO, Albandar HJ, Cohen PL (2015). Stat1 regulates lupus-like chronic graft-versus-host disease severity via interactions with Stat3. J Immunol.

[15] Eisenberg R (2003). The chronic graft-versus-host model of systemic autoimmunity. Curr Dir Autoimmun.

[16] Shao WH, Eisenberg RA, Cohen PL (2008). The Mer receptor tyrosine kinase is required for the loss of B cell tolerance in the chronic graft-versus-host disease model of systemic lupus erythematosus. J Immunol.

[17] Choudhury A, Maldonado MA, Cohen PL, Eisenberg RA (2005). The role of host CD4 T cells in the pathogenesis of the chronic graft-versus-host model of systemic lupus erythematosus. J Immunol.

[18] Eisenberg RA, Via CS (2012). T cells, murine chronic graft-versus-host disease and autoimmunity. J Autoimmun.

[19] Wong CK, Lit LC, Tam LS, Li EK, Wong PT, Lam CW (2008). Hyperproduction of IL-23 and IL-17 in patients with systemic lupus erythematosus: implications for Th17-mediated inflammation in auto-immunity. Clin Immunol.

[20] Shao WH, Zhen Y, Finkelman FD, Cohen PL (2014). The Mertk receptor tyrosine kinase promotes T-B interaction stimulated by IgD B-cell receptor cross-linking. J Autoimmun.

[21] Karantanos T, Chistofides A, Barhdan K, Li L, Boussiotis VA (2016). Regulation of T cell differentiation and function by EZH2. Front Immunol.

[22] He S, Wang J, Kato K, Xie F, Varambally S, Mineishi S, Kuick R, Mochizuki K, Liu Y, Nieves E (2012). Inhibition of histone methylation arrests ongoing graft-versus-host disease in mice by selectively inducing apoptosis of alloreactive effector T cells. Blood.

[23] Velichutina I, Shaknovich R, Geng H, Johnson NA, Gascoyne RD, Melnick AM, Elemento O (2010). EZH2-mediated epigenetic silencing in germinal center B cells contributes to proliferation and lymphomagenesis. Blood.

[24] Zingg D, Arenas-Ramirez N, Sahin D, Rosalia RA, Antunes AT, Haeusel J, Sommer L, Boyman O (2017). The histone methyltransferase Ezh2 controls mechanisms of adaptive resistance to tumor immunotherapy. Cell Rep.

[25] Davidson A, Aranow C (2006). Pathogenesis and treatment of systemic lupus erythematosus nephritis. Curr Opin Rheumatol.

[26] Patel DR, Richardson BC (2010). Epigenetic mechanisms in lupus. Curr Opin Rheumatol.

[27] Rohraff DM, He Y, Farkash EA, Schonfeld M, Tsou PS, Sawalha AH. Inhibition of EZH2 ameliorates lupus-like disease in MRL/lpr mice. Arthritis Rheumatol. 2019;71(10):1681–90. 10.1002/art.40931 PMC6764871 31106974

[28] Tsou PS, Coit P, Kilian NC, Sawalha AH (2018). EZH2 modulates the DNA methylome and controls T cell adhesion through Junctional adhesion molecule a in lupus patients. Arthritis Rheumatol.

[29] Miranda TB, Cortez CC, Yoo CB, Liang G, Abe M, Kelly TK, Marquez VE, Jones PA (2009). DZNep is a global histone methylation inhibitor that reactivates developmental genes not silenced by DNA methylation. Mol Cancer Ther.

[30] Mayr C, Wagner A, Stoecklinger A, Jakab M, Illig R, Berr F, Pichler M, Di Fazio P, Ocker M, Neureiter D (2015). 3-Deazaneplanocin a may directly target putative cancer stem cells in biliary tract cancer. Anticancer Res.

[31] Macanovic M, Sinicropi D, Shak S, Baughman S, Thiru S, Lachmann PJ (1996). The treatment of systemic lupus erythematosus (SLE) in NZB/W F1 hybrid mice; studies with recombinant murine DNase and with dexamethasone. Clin Exp Immunol.

[32] Fouse SD, Costello JF (2013). Cancer stem cells activate STAT3 the EZ way. Cancer Cell.

[33] Harada T, Kyttaris V, Li Y, Juang YT, Wang Y, Tsokos GC (2007). Increased expression of STAT3 in SLE T cells contributes to enhanced chemokine-mediated cell migration. Autoimmunity.

[34] Chen SY, Liu MF, Kuo PY, Wang CR (2019). Upregulated expression of STAT3/IL-17 in patients with systemic lupus erythematosus. Clin Rheumatol.

[35] Katsuyama T, Tsokos GC, Moulton VR (2018). Aberrant T cell signaling and subsets in systemic lupus erythematosus. Front Immunol.

[36] Hedrich CM, Rauen T, Apostolidis SA, Grammatikos AP, Rodriguez Rodriguez N, Ioannidis C, Kyttaris VC, Crispin JC, Tsokos GC (2014). Stat3 promotes IL-10 expression in lupus T cells through trans-activation and chromatin remodeling. Proc Natl Acad Sci U S A.

[37] Dobenecker MW, Park JS, Marcello J, McCabe MT, Gregory R, Knight SD, Rioja I, Bassil AK, Prinjha RK, Tarakhovsky A (2018). Signaling function of PRC2 is essential for TCR-driven T cell responses. J Exp Med.

